# Efficient Force Control Learning System for Industrial Robots Based on Variable Impedance Control

**DOI:** 10.3390/s18082539

**Published:** 2018-08-03

**Authors:** Chao Li, Zhi Zhang, Guihua Xia, Xinru Xie, Qidan Zhu

**Affiliations:** College of Automation, Harbin Engineering University, Harbin 150001, China; li_chao@hrbeu.edu.cn (C.L.); xiaguihua@hrbeu.edu.cn (G.X.); xiexinru@hrbeu.edu.cn (X.X.); zhuqidan@hrbeu.edu.cn (Q.Z.)

**Keywords:** force control, variable impedance control, efficient learning, Gaussian processes, industrial robot

## Abstract

Learning variable impedance control is a powerful method to improve the performance of force control. However, current methods typically require too many interactions to achieve good performance. Data-inefficiency has limited these methods to learn force-sensitive tasks in real systems. In order to improve the sampling efficiency and decrease the required interactions during the learning process, this paper develops a data-efficient learning variable impedance control method that enables the industrial robots automatically learn to control the contact force in the unstructured environment. To this end, a Gaussian process model is learned as a faithful proxy of the system, which is then used to predict long-term state evolution for internal simulation, allowing for efficient strategy updates. The effects of model bias are reduced effectively by incorporating model uncertainty into long-term planning. Then the impedance profiles are regulated online according to the learned humanlike impedance strategy. In this way, the flexibility and adaptivity of the system could be enhanced. Both simulated and experimental tests have been performed on an industrial manipulator to verify the performance of the proposed method.

## 1. Introduction

With the development of the modern robotics, compliance control is becoming an important component for industrial robots. Control of contact force is crucial for successfully executing operational tasks that involve physical contacts, such as grinding, deburring, or assembly. In the structured environment, good performances could be achieved using classical force control methods [1]. However, it is difficult to control the contact force effectively in the unstructured environment. Impedance control [2] provides a suitable control architecture for robots in both unconstrained and constrained motions by establishing a suitable mass-spring-damper system.

Neuroscience studies have demonstrated how humans perform specific tasks by adapting muscle stiffness [3]. Kieboom [4] studied the impedance regulation rule for bipedal locomotion and found that variable impedance control can improve gait quality and reduce energy expenditure. The ability to task-dependently change the impedance is one important aspect of biomechanical systems that leads to its good performance. Recently, many researchers have explored the benefits of varying the impedance during the task for robotics [5,6,7,8,9]. The basic idea is to adjust the impedance parameters according to the force feedback. Humanlike adaptivity was achieved in [9] by adapting force and impedance, providing an intuitive solution for human-robot interactions. Considering ensuring safe interaction, Calinon [6] proposed a learning-based control strategy with variable stiffness to reproduce the skill characteristics. Kronander [10] has demonstrated the stability of variable impedance control for the control system. Variable impedance not only enables control of the dynamic relationship between contact forces and robot movements, but also enhances the flexibility of the control system. Generally, the methods of specifying the varying impedance can be classified into three categories.

(1) Optimal control. The basic idea is to dynamically adjust the gains by the feedback information using optimization techniques. They are usually robust to uncertain systems. Medina [11] proposed a variable compliance control approach based on risk-sensitive optimal feedback control. This approach has the benefits of high adaptability to uncertain and variable environment. The joint torque and the joint stiffness are independently and optimally modulated using the optimal variable stiffness control in [12]. Adaptive variable impedance control is proposed in [13], it stabilizes the system by adjusting the gains online according to the force feedback. To guarantee stable execution of variable impedance tasks, a tank-based approach to passive varying stiffness is proposed in [14].

(2) Imitation of human impedance. Humans have a perfect ability to complete a variety of interaction tasks in various environments by adapting their biomechanical impedance characteristics. These excellent abilities are developed over years of experience and stored in the central nervous system [15]. For the purpose of imitating the human impedance modulation manner, some methods have been proposed [6,8]. Toshi [16] discussed the impedance regulation law of the human hand during dynamic-contact tasks and proposed a bio-mimetic impedance control for robot. Lee [17] designed a variable stiffness control scheme imitating human control characteristics, and it achieved force tracking by adjusting the target stiffness without estimating the environment stiffness. Yang [18] introduced a coupling interface to naturally transfer human impedance adaptive skill to the robot by demonstration. Kronander [19] and Li [20] addressed the problem of compliance adjusting in a robot learning from demonstrations (RLfD), in which a robot could learn to adapt the stiffness based on human–robot interaction. Yang [21] proposed a framework for learning and generalizing humanlike variable impedance skills, combining the merits of the electromyographic (EMG) and dynamic movement primitives (DMP) model. To enhance the control performance, the problem of transferring human impedance behaviors to the robot has been studied in [22,23,24]. These methods usually use the EMG device to collect the muscle activities information, based on which variation of human limb impedance can be estimated.

(3) Reinforcement learning (RL). Reinforcement learning constitutes a significant aspect of the artificial intelligence field with numerous applications ranging from medicine to robotics [25,26]. Researchers have recently focused on learning an appropriate modulation strategy by means of RL to adjust the impedance characteristic of robot [7,27,28,29]. Du [30] proposed a variable admittance control method based on fuzzy RL for human–robot interaction. It improves the positioning accuracy and reduces the required energy by dynamically regulating the virtual damping. Li [31] proposed an BLF-based adaptive impedance control framework for a human–robot cooperation task. The impedance parameters were learned using the integral RL to get adaptive robot behaviors.

In summary, to derive an effective variable impedance controller, the first category and the second category of methods usually need advanced engineering knowledge about the robot and the task, as well as designing these parameters. Learning variable impedance control based on RL is a promising and powerful method, which can get the proper task-specific control strategy automatically through trial-and-error. RL algorithms can be broadly classified as two types: model-free and model-based. In model-free RL, policy is found without even building a model of the dynamics, and the policy parameters can be searched directly. However, for each sampled trajectory, it is necessary to interact with the robot, which is time-consuming and expensive. In model-based RL, the algorithm explicitly builds a transition model of the system, which is then used for internal simulations and predictions. The (local) optimal policy is improved based on the evaluations of these internal simulations. The model-based RL is more data-efficient than the model-free RL, but more computationally intensive [32,33]. In order to extend to high-dimensional tasks conveniently and avoid the model-bias of model-based RL algorithm, the existing learning variable impedance control methods are most commonly based on a model-free RL algorithm. Buchli [5] proposed a novel variable impedance control based on PI^2^, which is a model-free RL algorithm. It realized simultaneous regulation of motion trajectory and impedance gains using DMPs. This algorithm has been successfully extended to high-dimensional robotic tasks such as opening door, picking up pens [34], box flipping task [35] and sliding switch task for tendon-driven hand [36]. Stulp [29] further studied the applicability of PI^2^ in stochastic force field, and it was able to find motor policies that qualitatively replicate human movement. Considering the coupling between degrees of freedom (DoFs), Winter [37] developed a C-PI^2^ algorithm based on PI^2^. Its learning speed was much higher than that of previous algorithms.

However, these methods usually require hundreds or thousands of rollouts to achieve satisfactory performance, which is unexpected for a force control system. None of these works address the issue of sampling efficiency. Improving data-efficiency is critical to learning to perform force-sensitive tasks, such as operational tasks of fragile components, because too many physical interactions with the environment during the learning process is usually infeasible. Alternatively, model-based RL is a promising way to improve the sampling efficiency. Fast convergence towards an optimal strategy could be guaranteed. Shadmehr [38] demonstrated that humans learn an internal model of the force field and compensate for external perturbations. Franklin [39] presented a model which combines three principles to learn stable, accurate, and efficient movements. It is able to accurately model empirical data gathered in force field experiments. Koropouli [27] investigated the generalization problem of force control policy. The force-motion mapping policy was learned from a set of demonstrated data to endow robots with certain human-like adroitness. Mitrovic [28] proposed to learn both the dynamics and the noise properties through supervised learning, using locally weighted projection regression. This model was then used in a model-based stochastic optimal controller to control a one-dimensional antagonistic actuator. It improved the accuracy performance significantly, but the analytical dynamic model still had accuracy limitations. These methods did not solve the key problem of model bias well.

Therefore, in order to improve the sampling efficiency, we propose a data-efficient learning variable impedance control method based on model-based RL that enables the industrial robots to automatically learn to control the contact force in the unstructured environment. A probabilistic Gaussian process (GP) model is approximated as a faithful proxy of the transition dynamics of the system. Then the probabilistic model is used for internal system simulation to improve the data-efficiency by predicting the long-term state evolution. This method reduces the effects of model-bias effectively by incorporating model uncertainty into long-term planning. The impedance profiles are regulated automatically according to the (sub)optimal impedance control strategy learned by the model-based RL algorithm to track the desired contact force. Additionally, we present a way of taking the penalty of control actions into account during planning to achieve the desired impedance characteristics. The performances of the system are verified through simulations and experiments on a six-DoF industrial manipulator. This system outperforms other learning variable impedance control methods by at least one order of magnitude in terms of learning speed.

The main contributions of this paper can be summarized as follows: (1) A data-efficient learning variable impedance control method is proposed to improve the sampling efficiency, which could significantly reduce the required physical interactions with environment during force control learning process. (2) The proposed method learns an impedance regulation strategy, based on which the impedance profiles are regulated online in a real-time manner to track the desired contact force. In this way, the flexibility and adaptivity of compliance control could be enhanced. (3) The impedance strategy with humanlike impedance characteristics is learned automatically through continuous explorations. There is no need to use additional sampling devices, such as EMG electrodes, to transfer human skills to the robot through demonstrations.

## 2. System Model and Contact Force Observer

### 2.1. Interaction Model

**Figure 1:**
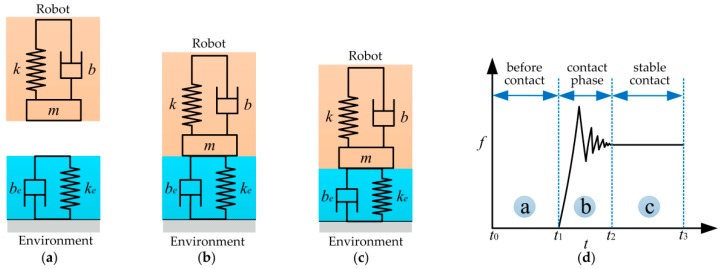
The interaction model of the system. (**a**) Without any contact between the robot and the environment; (**b**) critical point when contact occurs; (**c**) stable contact with the environment; (**d**) contact force diagram when robot comes in contact with the environment.

When the robot interacts with the rigid environment, the robot could be presented by a second order mass-spring-damper system, and the environment could be modeled as a spring-damping model with stiffness $k_{e}$ and damping $b_{e}$. The interaction model of the system is illustrated in Figure 1. Figure 1d shows a contact force diagram when robot comes in contact with the environment. m, b, and *k* denote the mass, damping, and stiffness of the robot end-effector, respectively. Let f be the contact force applied by the robot to the environment once a contact between both is established.

The contact process between the robot and the environment can be divided into three phases. In the first phase, the robot is approaching toward the environment (Figure 1a). There is no contact during this phase, and the contact force is zero (Figure 1d, $t_{0}$ − $t_{1}$). In the second phase, the robot is in contact with the environment (Figure 1b). During this phase, the contact force increases rapidly (Figure 1d, $t_{1}$ − $t_{2}$). Collision is inevitable, and the collision is transient and strongly nonlinear. In the third phase, the robot contacts with the environment continuously (Figure 1c) and the contact force is stabilized to the desired value (Figure 1d, $t_{2}$ − $t_{3}$).

High values of contact force are generally undesirable since they may stress both the robot and the manipulated object. Therefore, the overshoot and the oscillation caused by the inevitable collision should be suppressed effectively.

### 2.2. Contact Force Observer Based on Kalman Filter

The contact force is the quantity describing the state of interaction in the most complete fashion. To this end, the availability of force measurements is expected to provide enhanced performance for controlling interaction [1]. Recently, several methods have been proposed to make the force control possible without dedicated sensors, such as virtual force sensor [40] and contact force estimation methods based on motor signals [41,42]. However, to realize precise force control, industrial robots are typically equipped with F/T sensors at the wrist to measure the contact force. The measurements of force sensors do not correspond to the actual environmental interaction forces which usually contain inertial force and gravity. Moreover, the raw signals sampled from the force sensor may be corrupted by noise, especially in the industrial environment where equipped with large equipment. The electromagnetic noise, vibration noise, electrostatic effect, and thermal noise are very strong and complex. These disturbances seriously affect the measurement of the F/T sensor which will degrade the quality of force control. Hence, a contact force observer based on the Kalman filter is designed to estimate the actual contact force and moment applied at the contact point.

**Figure 2:**
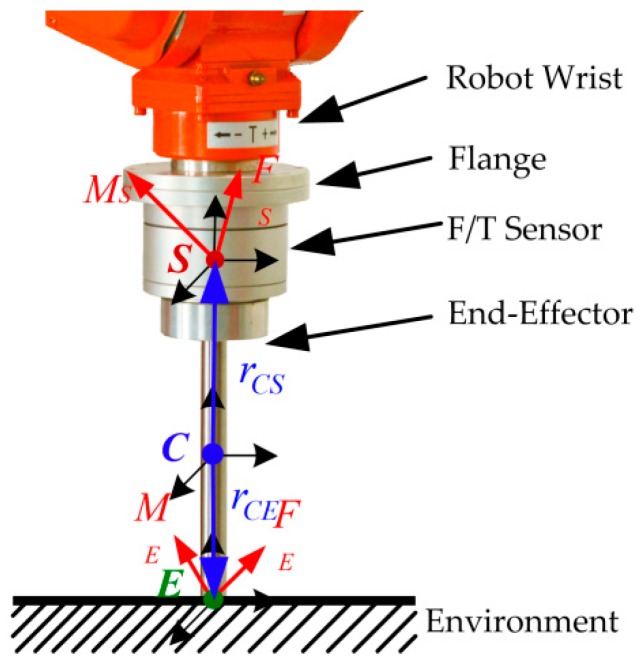
Contact force and moment applied at the end-effector.

Figure 2 illustrates the contact force and moment applied at the end-effector. The center of mass of the end-effector locates at *C*. *E* is the contact point between the end-effector and the environment. The center of mass of the F/T sensor is *S*. As shown in Figure 2, the corresponding coordinate frames with the principal axes are denoted by $\Sigma_{C}$, $\Sigma_{E}$, and $\Sigma_{S}$, respectively. The world frame is denoted by $\Sigma_{W}$.

Assume that the robot moves slowly during the tasks, the inertial effect could be negligible. The contact force model could be built using the Newton–Euler equations [43]

(1) $$\left[\begin{array}{cc} R_{E}^{C} & 0\\ S(r_{CE}^{C})R_{E}^{C} & R_{E}^{C} \end{array}\right]\left[\begin{array}{c} F_{E}^{E}\\ M_{E}^{E} \end{array}\right]=\left[\begin{array}{cc} R_{S}^{C} & 0\\ S(r_{CS}^{C})R_{S}^{C} & R_{S}^{C} \end{array}\right]\left[\begin{array}{c} F_{S}^{S}\\ M_{S}^{S} \end{array}\right]-\left[\begin{array}{c} mR_{W}^{C}\\ 0 \end{array}\right]g^{W},$$

where the matrix $R_{j}^{i}$ is the rotation matrix of frame $\Sigma_{j}$ with respect to frame $\Sigma_{i}$. $F_{E}^{E}$ and $M_{E}^{E}$ are the actual contact forces and moments applied at the end-effector. $F_{S}^{S}$ and $M_{S}^{S}$ are the measured forces and moments by the F/T sensor. m is the mass of the end-effector. *g^W^* is the gravitational acceleration. $r_{CE}^{C}$ denotes the vector from *C* to *E* with respect to frame $\Sigma_{C}$. $r_{CS}^{C}$ is the vector from *C* to *S* with respect to frame $\Sigma_{C}$. The skew-symmetry operator applied to vector b is donated as $S\left(b\right)=S_{b}$. To define the state vector x and the measurement vector y

(2) $$x={\left[\begin{array}{cccc} F_{S}^{S} & M_{S}^{S} & {\dot{F}}_{S}^{S} & {\dot{M}}_{S}^{S} \end{array}\right]}^{T},$$

(3) $$y={\left[\begin{array}{cc} F_{E}^{E} & M_{E}^{E} \end{array}\right]}^{T},$$

**Figure 3:**
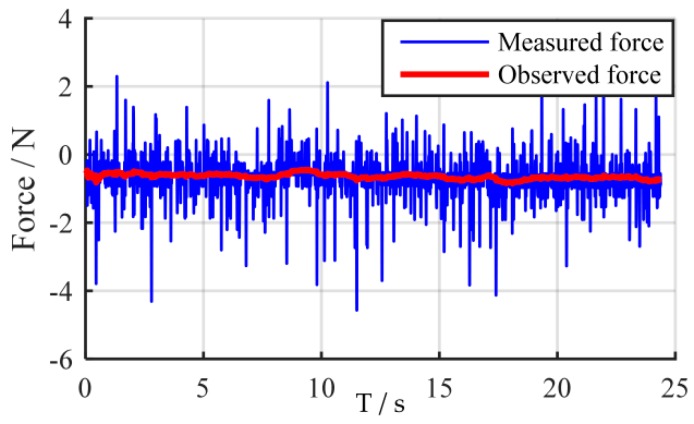
An implementation example of the contact force observer.

According to (1), the system model and measurement model can be established

(4) $$\begin{array}{l} \dot{x}(t)=A_{0}x(t)+w_{x}\\ y(t)=H_{0}x(t)+D_{0}g^{W}+v_{y} \end{array},$$

where $A_{0}=\left[\begin{array}{cc} 0_{6\times 6} & I_{6\times 6}\\ 0_{6\times 6} & 0_{6\times 6} \end{array}\right],$ $H_{0}=\left[\begin{array}{cccc} R_{S}^{E} & 0_{3\times 3} & 0_{3\times 3} & 0_{3\times 3}\\ R_{C}^{E}[S(r_{CS}^{C})-S(r_{CE}^{C})]R_{S}^{C} & R_{S}^{E} & 0_{3\times 3} & 0_{3\times 3} \end{array}\right],$ $D_{0}=m\left[\begin{array}{c} -R_{W}^{E}\\ R_{C}^{E}S(r_{CE}^{C})R_{W}^{C} \end{array}\right]$. $\omega_{x}$ and $v_{y}$ are the process noise and measurement noise. They are assumed to be independent of each other with normal probability distribution $p\left(\omega_{x}\right)\sim N\left(0,Q\right)$, $p\left(v_{y}\right)\sim N\left(0,R\right)$. Q, R are the corresponding covariance matrices which are assumed as diagonal matrices with constant diagonal elements. The model (4) can be discretized resulting in the discrete-time linear system

(5) $$\begin{array}{l} x_{k}=Ax_{k-1}+w_{k-1},\\ y_{k}=Hx_{k}+Dg^{W}+v_{k}, \end{array}$$

where $A=\left[\begin{array}{cc} I_{6\times 6} & I_{6\times 6}\\ 0_{6\times 6} & I_{6\times 6} \end{array}\right]$, $H=H_{0}$, $D=D_{0}$. The Kalman filter update consists of two steps:

1. Predict the state estimate ${\hat{x}}_{k|k-1}^{-}$ and the error covariance $P_{k|k-1}^{-}$ at time step *k* based on the results from the previous time step *k* − 1:
  (6) $$\begin{array}{l} {\hat{x}}^{-}{}_{k|k-1}=A{\hat{x}}_{k-1},\\ P_{k|k-1}^{-}=AP_{k-1}A^{T}+Q. \end{array}$$
2. Correct the predictions ${\hat{x}}_{k}$ based on the measurements $y_{k}$:
  (7) $$\begin{array}{l} {\hat{x}}_{k}={\hat{x}}_{k|k-1}^{-}+K_{k}(y_{k}-H{\hat{x}}_{k|k-1}^{-}),\\ K_{k}=P_{k|k-1}^{-}H^{T}{\left(HP_{k|k-1}^{-}H+R\right)}^{-1},\\ P_{k}=(I-K_{k}H)P_{k|k-1}^{-}, \end{array}$$
  where $K_{k}$ is the Kalman gain, $P_{k}$ is the updated error covariance. In practice, the covariance matrices Q and R can automatically be calibrated based on the offline experimental data [41]. It should be mentioned that the larger the weights of Q are chosen, the more the observer will rely on the measurements. Larger diagonal elements in Q result in faster response time of the corresponding estimates, but this also results in increased noise amplification. An implementation example of the contact force observer is shown in Figure 3.

## 3. Position-Based Impedance Control for Force Control

One possible approach to achieve compliant behavior for robotics is the classical impedance control [2], which sometimes is also called force-based impedance control. In typical implementations, the controller has the positions as inputs and gives the motor torques as outputs. Force-based impedance control has been widely studied [44]. However, most commercial industrial robots emphasize the accuracy of trajectory following, and do not provide joint torque or motor current interfaces for users. Therefore, force-based impedance control is impossible on these robots.

**Figure 4:**
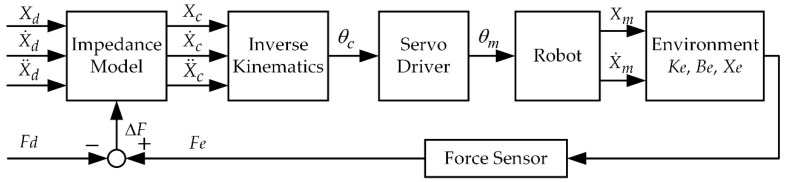
The position-based impedance control schematic.

Alternatively, another possible approach, which is typically suited for industrial robots, is the concept of admittance control [45], sometimes also called position-based impedance control. It maps from generalized forces to generalized positions. This control structure consists of an inner position control loop and an outer indirect force control loop. In constrained motion, the contact force measured by the F/T sensor modifies the desired trajectory in the outer impedance controller loop resulting in the compliant desired trajectory, which is to be tracked by the inner position controller. The position-based impedance control schematic for force control is shown in Figure 4. $X_{d}$, $X_{c}$, and $X_{m}$ denote the reference position trajectory, the commanded position trajectory which is sent to the robot, and the measured position trajectory, respectively. Assuming good tracking performance of the inner position controller for slow motions, the commanded trajectory is equal to the measured trajectory, i.e., $X_{m}=X_{c}$.

Typically, the impedance model is chosen as a linear second order system

(8) $$M_{d}({\ddot{X}}_{c}-{\ddot{X}}_{d})+B_{d}({\dot{X}}_{c}-{\dot{X}}_{d})+K_{d}(X_{c}-X_{d})=F_{e}-F_{d},$$

where $M_{d}$, $B_{d}$, and $K_{d}$ are, respectively, the target inertial, damping, and stiffness matrices. $F_{d}$ is the desired contact force. $F_{e}$ is the actual contact force. The transfer-function of the impedance model is

(9) $$H(s)=\frac{E(s)}{\Delta F(s)}=\frac{1}{M_{d}s^{2}+B_{d}s+K_{d}},$$

where $\Delta F=F_{e}-F_{d}$ denotes the force tracking error. $E=X_{c}-X_{d}$ is the desired position increment, and it is used to modify the reference position trajectory $X_{d}$ to produce the commanded trajectory $X_{c}=X_{d}+E$, which is then tracked by the servo driver system. 

To compute the desired position increment, discretize (9) using bilinear transformation

(10) $$H(z)=H(s)|{}_{s=\frac{2}{T}\frac{z-1}{z+1}}=\frac{T^{2}{\left(z+1\right)}^{2}}{\omega_{1}z^{2}+\omega_{2}z+\omega_{3}},$$

(11) $$\begin{array}{l} \omega_{1}=4M_{d}+2B_{d}T+K_{d}T^{2},\\ \omega_{2}=-8M_{d}+2K_{d}T^{2},\\ \omega_{3}=4M_{d}-2B_{d}T+K_{d}T^{2}. \end{array}$$

Here, T is the control cycle. The desired position increment at time n is derived as

(12) $$E(n)=\omega_{1}^{-1}\{T^{2}[\Delta F(n)+2\Delta F(n-1)+\Delta F(n-2)]\;-\omega_{2}\delta X(n-1)-\omega_{3}\delta X(n-2)\}.$$

The environment is assumed to be a spring-damping model with stiffness $K_{e}$ and damping $B_{e}$. $X_{e}$ is the location of the environment. The contact force between the robot and the environment is then simplified as

(13) $$F_{e}=B_{e}({\dot{X}}_{c}-{\dot{X}}_{e})+K_{e}(X_{c}-X_{e}).$$

Replacing $X_{d}$ in (8) with the initial environment location $X_{e}$, and substituting (13) into (8), the new impedance equation is then converted to

(14) $$M_{d}({\ddot{X}}_{c}-{\ddot{X}}_{e})+(B_{d}+B_{e})({\dot{X}}_{c}-{\dot{X}}_{e})\;+(K_{d}+K_{e})(X_{c}-X_{e})=F_{d}.$$

Due to the fact that it is difficult to obtain accurate environment information. Therefore, if the environment stiffness $K_{e}$ and damping $B_{e}$ change, the dynamic characteristics of the system will change consequently. To guarantee the dynamic performance, the impedance parameters $\left[M_{d},B_{d},K_{d}\right]$ should be adjusted correspondingly.

## 4. Data-Efficient Learning Variable Impedance Control with Model-Based RL

Generally, too many physical interactions with the environment are infeasible for learning to execute force-sensitive tasks. In order to reduce the required interactions with the environment during force control learning process, a learning variable impedance control approach with model-based RL algorithm is proposed in the following to learn the impedance regulation strategy.

### 4.1. Scheme of the Data-Efficient Learning Variable Impedance Control

**Figure 5:**
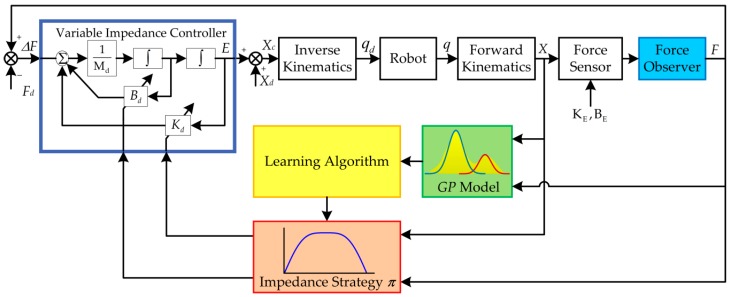
Scheme of the data-efficient learning variable impedance control.

The scheme of the method is illustrated in Figure 5. $F_{d}$ is the desired value of contact force, F is the actual contact force estimated by the force observer, and ΔF is the force tracking error. The desired position increment of the end-effector E is calculated using the variable impedance controller. $X_{d}$ is the desired reference trajectory. The desired joints positions $q_{d}$ are calculated by adopting inverse kinematics according to the commanded trajectory $X_{c}$. The actual Cartesian position of the end-effector X could be achieved using the measured joints positions q by means of forward kinematics. The joints are controlled by the joint motion controller of the industrial robot. $K_{E}$ and $B_{E}$ denote the unknown stiffness and damping of the environment, respectively.

The transition dynamics of the system is approximated by the GP model which is trained using the collected data. Then the learning algorithm is used to learn the (sub)optimal impedance control strategy π while predicting the system evolution using the GP model. Instead of the motion trajectory, the proposed method learns an impedance regulation strategy, based on which the impedance profiles are regulated online in a real-time manner to control the contact force. In this way, the dynamic relationship between contact force and robot movement could be controlled in a continuous manner. Moreover, the flexibility and adaptivity for compliance control could be enhanced. Positive definite impedance parameters could ensure the asymptotic stability of the original desired dynamics, but it is not recommended to modify the target inertia matrix because it is easy to cause the system to instability [45]. To simplify the calculation, the target inertial matrix is chosen as $M_{d}=I$. Consequently, the target stiffness $K_{d}$ and the damping $B_{d}$ are the parameters that should be tuned in variable impedance control. Based on the learned impedance strategy, the impedance parameters $u=\left[K_{d}\;B_{d}\right]$ are calculated according to the states of the contact force and the position of the end-effector, which are then transferred to the variable impedance controller.

The learning process of variable impedance strategy consists of seven main steps:

1. Initializing the strategy parameters stochastically, applying the random impedance parameters to the system and recording the sampled data.
2. Training the system transition dynamics, i.e., the GP model, using all historical data.
3. Inferring and predicting the long-term evolution of the states according to the GP model.
4. Evaluating the total expected cost $J^{\pi }\left(\theta \right)$ in T steps, and calculating the gradients of the cost $dJ^{\pi }\left(\theta \right)/d\theta$ with respect to the strategy parameters θ.
5. Learning the optimal strategy $\pi^{*}\leftarrow \pi \left(\theta \right)$ using the gradient-based policy search algorithm.
6. Applying the impedance strategy to the system, then executing a force tracking task using the learned variable impedance strategy and recording the sampled data simultaneously.
7. Repeating steps (2)–(6) until the performance of force control is satisfactory.

### 4.2. Variable Impedance Strategy

The impedance control strategy is defined as π:x↦u=π(x,θ), where the inputs of the strategy are the observed states of the robot $x=\left[X\;F\right]\in {\mathbb{R}}^{D}$, the outputs of the strategy are target stiffness *K_d_* and damping *B_d_* which can be written as matrix $u=\left[K_{d}\;B_{d}\right]\in {\mathbb{R}}^{F}$, and θ are the strategy parameters that to be learned. Here, the GP controller is chosen as the control strategy

(15) $$\pi_{t}=\pi (x_{t},\theta )=\sum_{i=1}^{n}\beta_{\pi ,i}k(x_{\pi },x_{t})=\beta_{\pi }^{T}K(X_{\pi },x_{t}),$$

(16) $$\beta_{\pi }={\left(K_{\pi }(X_{\pi },X_{\pi })+\sigma_{\varepsilon ,\pi }^{2}I\right)}^{-1}y_{\pi },$$

(17) $$k(x_{\pi },x_{t})=\sigma_{f,\pi }^{2}\exp(-\frac{1}{2}{\left(x_{\pi i}-x_{t}\right)}^{T}\Lambda^{-1}(x_{\pi i}-x_{t})),$$

where $x_{t}$ is the test input. $X_{\pi }=\left[x_{\pi 1},\ldots ,x_{\pi n}\right]$ are the training inputs, and they are the centers of the Gaussian basis functions. *n* is the number of the basis functions. $y_{\pi }$ is the training targets, which are initialized to values close to zero. *K* is the covariance matrix with entries $K_{ij}=k\left(x_{i},x_{j}\right)$. $\Lambda =diag\left(l_{1}^{2},\ldots ,l_{D}^{2}\right)$ is the length-scale matrix where $l_{i}$ is the characteristic length-scale of each input dimension, $\sigma_{f,\pi }^{2}$ is the signal variance, which is fixed to one here, $\sigma_{\varepsilon ,\pi }^{2}$ is the measurement noise variance, and $\theta =\left[X_{\pi },y_{\pi },l_{1},\ldots ,l_{D},\sigma_{f,\pi }^{2},\sigma_{\varepsilon ,\pi }^{2}\right]$ is the hyper-parameters of the controller. Using the GP controller, more advanced nonlinear tasks could be performed thanks for its flexibility and smoothing effect. Obviously, the GP controller is functionally equivalent to a regularized RBF network if $\sigma_{f,\pi }^{2}=1$ and $\sigma_{\varepsilon ,\pi }^{2}\neq 0$. The impedance parameters are calculated in real-time according to the impedance strategy π and the states $x_{t}$. The relationship between the impedance parameters and the control strategy can be written as

(18) $$[\begin{array}{cc} K_{d} & B_{d} \end{array}]=u=\pi (x_{t},\theta )=\beta_{\pi }^{T}K(X_{\pi },x_{t}).$$

In practical systems, the physical limits of the impedance parameters should be considered. The preliminary strategy π should be squashed coherently through a bounded and differentiable saturation function. The saturation function has to be on a finite interval, such that a maximum and minimum are obtained for finite function inputs. Furthermore, the function should be monotonically increasing. The derivative and second derivative of the saturation function have to be zero at the boundary points to require stationary points at these boundaries. Specifically, consider the third-order Fourier series expansion of a trapezoidal wave κ(x)=[9sin(x)+sin(3x)]/8, which is normalized to the interval [−1, 1]. Given the boundary conditions, the saturation function is defined as

(19) $$S(\pi_{t})=u_{\min}+u_{\max}+u_{\max}\frac{9\sin\pi_{t}+\sin(3\pi_{t})}{8}.$$

If the function is considered on the domain [3π/2, 2π], the function is monotonically increasing, and the control signal *u* is squashed to the interval $\left[u_{\min}\;u_{\min}+u_{\max}\right]$.

### 4.3. Probabilistic Gaussian Process Model

Transition models have a large impact on the performance of model-based RL, since the learned strategy inherently relies on the quality of the learned forward model, which essentially serves as a simulator of the system. The transition models that have been employed for model-based RL can be classified into two main categories [25]: the deterministic models and the stochastic models. Despite the intensive computation, the state-of-the-art approach for learning the transition models is the GP model [46], because it is capable of modeling a wide spread of nonlinear systems by explicitly incorporating model uncertainty into long-term planning, which is a key problem in model-based learning. In addition, the GP model shows good convergence properties which are necessary for implementation of the algorithm. A GP model can be thought as a probabilistic distribution over possible functions, and it is completely specified by a mean function m(⋅) and a positive semi-definite covariance function k(⋅,⋅), also called a kernel.

Here, we consider the unknown function that describes the system dynamics

(20) $$\begin{array}{l} x_{t}=f(x_{t-1},u_{t-1}),\\ y_{t}=x_{t}+\varepsilon_{t}, \end{array}$$

with continuous state inputs $x\in {\mathbb{R}}^{D}$, control inputs $u\in {\mathbb{R}}^{F}$, training targets $y\in {\mathbb{R}}^{E}$, unknown transition dynamics f, and i.i.d. system noise $\varepsilon \sim N\left(0,\sigma_{\varepsilon }^{2}\right)$. In order to take the model uncertainties into account during prediction and planning, the proposed approach does not make a certainty equivalence assumption on the learned model. Instead, it learns a probabilistic GP model and infers the posterior distribution over plausible function f from the observations. For computation convenience, we consider a prior mean m≡0 and the squared exponential kernel

(21) $$f(x)\sim GP(m(x),k(x,x^{\prime })),$$

(22) $$k(x,x^{\prime })=\alpha^{2}\exp\left(-\frac{1}{2}{\left(x-x^{\prime }\right)}^{T}\Lambda^{-1}(x-x^{\prime })\right)+\sigma_{\varepsilon }^{2}I,$$

where $\alpha^{2}$ is the variance of the latent function f, the weighting matrix $\Lambda =diag\left(\left[l_{1}^{2},\ldots l_{D}^{2}\right]\right)$ depends on the different characteristic length-scale $l_{i}$ of each input dimension. Given N training inputs $X=\left[x_{1},\ldots x_{N}\right]$ and corresponding training targets $y={\left[y_{1},\ldots y_{N}\right]}^{T}$, the GP hyper-parameters $\left[{\Lambda \;\alpha }^{2}{\;\sigma }_{\varepsilon }^{2}\right]$ could be learned using evidence maximization algorithm [46].

Given a deterministic test input $x_{*}$, the posterior prediction $p(f_{*}|x_{*})$ of the function value $f_{*}=f\left(x_{*}\right)$ is Gaussian distributed

(23) $$p(f_{*}|x_{*})\sim N(\mu_{*},\sum_{*}),$$

(24) $$\mu_{*}=m(x_{*})+k(x_{*},X){\left(K+\sigma_{\varepsilon }^{2}I\right)}^{-1}(y-m(X))=m(x_{*})+k(x_{*},X)\beta ,$$

(25) $$\sum_{*}=k(x_{*},x_{*})-k(x_{*},X){\left(K+\sigma_{\varepsilon }^{2}I\right)}^{-1}k(X,x_{*}),$$

where $\beta ={\left(K+\sigma_{\varepsilon }^{2}I\right)}^{-1}\left(y-m\left(X\right)\right)$, and K=k(X,X) is the kernel matrix.

In our force control learning system, the function of the GP model is defined as $f:{\mathbb{R}}^{D+F}\to {\mathbb{R}}^{E}$,$\left(x_{t-1},u_{t-1}\right)⟼\Delta_{t}=x_{t}-x_{t-1}+\delta_{t}$, where ${\hat{x}}_{t-1}=\left(x_{t-1},u_{t-1}\right)$ is the training input tuples. Take the state increments $\Delta_{t}=x_{t}-x_{t-1}+\delta_{t}$ as training targets, where $\delta_{t}\sim N\left(0,\Sigma_{\varepsilon }\right)$ is i.i.d. measurement noise. Since the state differences vary less than the absolute values, the underlying function that describes these differences varies less. Therefore, it implies that the learning process is easier and that less data is needed to find an accurate model. Moreover, when the predictions leave the training set, the prediction will not fall back to zero but remain constant.

### 4.4. Approximate Prediction for Strategy Evaluation

For the sake of reducing the required physical interactions with the robots while getting an effective control strategy, the effective utilization of sampled data must be increased. To this end, the learned probabilistic GP model is used as the faithfully dynamics of the system, which is then used for internal simulations and predictions about how the real system would behave. The (sub)optimal strategy is improved based on the evaluations of these internal virtual trials. Thus, the data-efficiency is improved.

**Figure 6:**
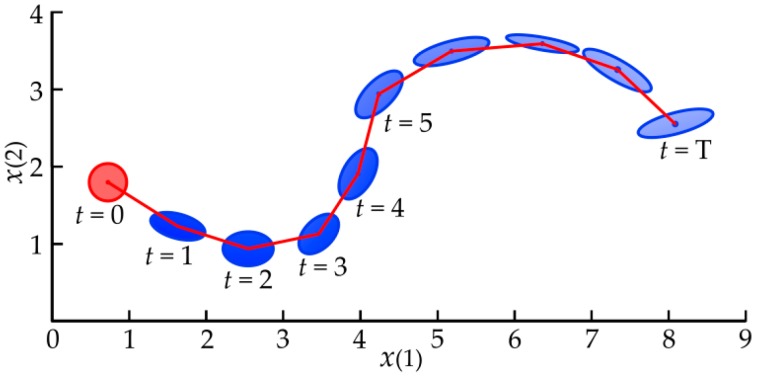
A conceptual illustration of long-term predictions of state evolution.

To evaluate the strategy, the long-term predictions of state $p\left(x_{1}\right),\ldots ,p\left(x_{T}\right)$ should be computed iteratively from the initial state distribution $p\left(x_{0}\right)$ by cascading one-step predictions [47]. Since the GP model can map the Gaussian-distributed states space to the targets space, the uncertainties of the inputs can pass through the model, and the uncertainties of the model are taken into account in the long-term planning. A conceptual illustration of long-term predictions of state evolution [48] is shown in Figure 6.

The one-step prediction of the states can be summarized as

(26) $$p(x_{t-1})\to p(u_{t-1})\to p(x_{t-1},u_{t-1})\to p(\Delta_{t})\to p(x_{t}).$$

As $u_{t-1}=\pi \left(x_{t-1}\right)$ is a function of state $x_{t-1}$ and $p(x_{t-1})$ is known, the calculation of $p(x_{t})$ requires a joint distribution $p\left({\hat{x}}_{t-1}\right)=p\left(x_{t-1},u_{t-1}\right)$. First, we calculate the predictive control signal $p(u_{t-1})$ and subsequently the cross-covariance $\mathrm{cov}\left[x_{t-1},u_{t-1}\right]$. Then, $p\left(x_{t-1},u_{t-1}\right)$ is approximated by a Gaussian distribution [47]

(27) $$p({\hat{x}}_{t-1})=p(x_{t-1},u_{t-1})=N({\hat{\mu }}_{t-1},{\hat{\sum }}_{t-1})=N\left(\left[\begin{array}{c} \mu_{x_{t-1}}\\ \mu_{u_{t-1}} \end{array}\right],\left[\begin{array}{cc} \sum_{x_{t-1}} & \sum_{x_{t-1},u_{t-1}}\\ \sum_{x_{t-1},u_{t-1}}^{T} & \sum_{u_{t-1}} \end{array}\right]\right).$$

The distribution of the training targets $\Delta_{t}$ are predicted as

(28) $$p(\Delta_{t})=\int p(f({\hat{x}}_{t-1})|{\hat{x}}_{t-1})p({\hat{x}}_{t-1})d{\hat{x}}_{t-1},$$

where the posterior predictive distribution of the transition dynamics $p\left(f({\hat{x}}_{t-1}\right)|{\hat{x}}_{t-1})$ could be calculated using the Formulas (23)–(25). Using moment matching [49], $p\left(\Delta_{t}\right)$ could be approximated as a Gaussian distribution $N\left(\mu_{\Delta },\Sigma_{\Delta }\right)$. Then, a Gaussian approximation to the desired state distribution $p\left(x_{t}\right)$ is given as

(29) $$p(x_{t}|{\hat{\mu }}_{t-1},{\hat{\sum }}_{t-1})\sim N(\mu_{t},\sum_{t}),$$

(30) $$\mu_{t}=\mu_{t-1}+\mu_{\Delta },$$

(31) $$\sum_{t}=\sum_{t-1}+\sum_{\Delta }+\mathrm{cov}[x_{t-1},\Delta_{t}]+\mathrm{cov}[\Delta_{t},x_{t-1}],$$

(32) $$\mathrm{cov}[x_{t-1},\Delta_{t}]=\mathrm{cov}[x_{t-1},u_{t-1}]\sum_{u}^{-1}\mathrm{cov}[u_{t-1},\Delta_{t}].$$

### 4.5. Gradient-Based Strategy Learning

The goal of the learning algorithm is to find the strategy parameters that minimize the total expected costs $\theta^{*}=\arg\;\min J^{\pi }\left(\theta \right)$. The search direction can be selected using the gradient information. The total expected cost $J^{\pi }\left(\theta \right)$ in T steps is calculated according to the state evolution

(33) $$J^{\pi }(\theta )=\sum_{t=0}^{T}E\left[c(x_{t})\right],x_{0}\sim N(\mu_{0},\sum_{0}),$$

(34) $$E[c_{t}]=\int c_{t}N(x_{t}|\mu_{t},\sum_{t})dx_{t}.$$

where $c\left(x_{t}\right)$ is the instantaneous cost at time t, and $E\left[c\left(x_{t}\right)\right]$ is the expected values of the instantaneous cost with respect to the predictive state distributions.

The cost function in RL usually penalizes the Euclidean distance from the current state to the target state, without considering other prior knowledge. However, in order to make robots with the ability of compliance, the control gains should not be high for practical interaction tasks. Generally, high gains will result in instability in stiff contact situations due to the inherent manipulator compliance, especially for an admittance-type force controller. In addition, low gains lead to several desirable properties of the system, such as compliant behavior (safety and/or robustness), lowered energy consumption, and less wear and tear. This is similar to the impedance regulation rules of humans. Humans learn a task-specific impedance regulation strategy that combines the advantages of high stiffness and compliance. The general rule of thumb thus seems to be “be compliant when possible; stiffen up only when the task requires it”. In other words, impedance increasing ensures tracking accuracy while impedance decreasing ensures safety.

To make the robots with these impedance characteristics, we present a way of taking the penalty of control actions into account during planning. The instantaneous cost function is defined

(35) $$c_{t}=c_{b}^{}(x_{t})+c_{e}^{}(u_{t}),$$

(36) $$c_{b}^{}(x_{t})=1-\exp(-\frac{1}{2\sigma_{c}^{2}}d{\left(x_{t},x_{t\arg et}\right)}^{2})\in [0,1],\;$$

(37) $$c_{e}^{}(u_{t})=c_{e}(\pi (x_{t}))=\zeta \cdot {\left(u_{t}/u_{\max}\right)}^{2}.\;$$

Here, $c_{b}\left(x_{t}\right)$ is the cost caused by the state error, denoted by a quadratic binary saturating function, which saturates at unity for large deviations to the desired target state. d(⋅) is the Euclidean distance between the current state $x_{t}$ to the target state $x_{target}$ and $\sigma_{c}$ is the width of the cost function. $c_{e}\left(u_{t}\right)$ is the cost caused by the control actions, i.e., the mean squared penalty of impedance gains. The suitable impedance gains could be reduced by punishing the control actions. ζ is the action penalty coefficient. $u_{t}$ is the current control signal, and $u_{max}$ is the maximum control signal amplitude.

The gradients of $J^{\pi }\left(\theta \right)$ with respect to the strategy parameters θ are given by

(38) $$\frac{dJ^{\pi }(\theta )}{d\theta }=\frac{d\sum_{t=0}^{T}E[c(x_{t})]}{d\theta }=\sum_{t=0}^{T}\frac{dE[c(x_{t})]}{d\theta }.$$

The expected immediate cost $E\left[c\left(x_{t}\right)\right]$ requires averaging with respect to the state distribution $p\left(x_{t}\right)\sim N\left(\mu_{t},\Sigma_{t}\right)$, where $\mu_{t}$ and $\Sigma_{t}$ are the mean and the covariance of $p\left(x_{t}\right)$, respectively. The derivative in Equation (38) can be written as

(39) $$\frac{dE[c(x_{t})]}{d\theta }=\frac{dE[c(x_{t})]}{dp(x_{t})}\frac{dp(x_{t})}{d\theta }=\frac{\partial E[c(x_{t})]}{\partial \mu_{t}}\frac{d\mu_{t}}{d\theta }+\frac{\partial E[c(x_{t})]}{\partial \sum_{t}}\frac{d\sum_{t}}{d\theta }.\;$$

Given $c\left(x_{t}\right)$, the item $\partial E\left[c\left(x_{t}\right)\right]/\partial \mu_{t}$ and $\partial E\left[c\left(x_{t}\right)\right]/\partial \Sigma_{t}$ could be calculated analytically. Then we will focus on the calculation of $d\mu_{t}/d\theta$ and ${d\Sigma }_{t}/d\theta$. Due to the computation sequence of (26), we know that the predicted mean $\mu_{t}$ and the covariance $\Sigma_{t}$ are functionally dependent on $p\left(x_{t-1}\right)\sim N\left(\mu_{t-1},\Sigma_{t-1}\right)$ and the strategy parameters θ through $\mu_{t-1}$. We thus obtain

(40) $$\frac{d\mu_{t}}{d\theta }=\frac{\partial \mu_{t}}{\partial p(x_{t-1})}\frac{dp(x_{t-1})}{d\theta }+\frac{\partial \mu_{t}}{\partial \theta }=\frac{\partial \mu_{t}}{\partial \mu_{t-1}}\frac{d\mu_{t-1}}{d\theta }+\frac{\partial \mu_{t}}{\partial \sum_{t-1}}\frac{d\sum_{t-1}}{d\theta }+\frac{\partial \mu_{t}}{\partial \theta },\;$$

(41) $$\frac{d\sum_{t}}{d\theta }=\frac{\partial \sum_{t}}{\partial p(x_{t-1})}\frac{dp(x_{t-1})}{d\theta }+\frac{\partial \sum_{t}}{\partial \theta }=\frac{\partial \sum_{t}}{\partial \mu_{t-1}}\frac{d\mu_{t-1}}{d\theta }+\frac{\partial \sum_{t}}{\partial \sum_{t-1}}\frac{d\sum_{t-1}}{d\theta }+\frac{\partial \sum_{t}}{\partial \theta },\;$$

(42) $$\frac{\partial \mu_{t}}{\partial \theta }=\frac{\partial \mu_{\Delta }}{\partial p(u_{t-1})}\frac{\partial p(u_{t-1})}{\partial \theta }=\frac{\partial \mu_{\Delta }}{\partial \mu_{u}}\frac{\partial \mu_{u}}{\partial \theta }+\frac{\partial \mu_{\Delta }}{\partial \sum_{u}}\frac{\partial \sum_{u}}{\partial \theta },\;$$

(43) $$\frac{\partial \sum_{t}}{\partial \theta }=\frac{\partial \sum_{\Delta }}{\partial p(u_{t-1})}\frac{\partial p(u_{t-1})}{\partial \theta }=\frac{\partial \sum_{\Delta }}{\partial \mu_{u}}\frac{\partial \mu_{u}}{\partial \theta }+\frac{\partial \sum_{\Delta }}{\partial \sum_{u}}\frac{\partial \sum_{u}}{\partial \theta }.\;$$

By repeated application of the chain-rule, the Equations (39)–(43) can be computed analytically. We omit further lengthy details here and refer to [47] for more information. Then the non-convex gradient-based optimization algorithm—e.g., conjugate gradient—can be applied to find the strategy parameters $\theta^{*}$ that minimize $J^{\pi }\left(\theta \right)$.

## 5. Simulations and Experiments

To verify the proposed force control learning system based on variable impedance control, a series of simulation and experiment studies on the Reinovo REBot-V-6R-650 industrial robot (Shenzhen Reinovo Technology CO., LTD, Shenzhen, China) are conducted and presented in this section. Reinovo REBot-V-6R-650 is a six-DoF industrial manipulator with a six-axis Bioforcen F/T sensor (Anhui Biofrcen Intelligent Technology CO., LTD, Hefei, China) mounted at the wrist. The F/T sensor is used to percept the contact force of the end-effector. The sensing range of the F/T sensor is $\pm 625\;N\;F_{x},F_{y}$, $\pm 1250\;N\;F_{z}$, $\pm 25\;\mathrm{Nm}\;T_{x},T_{y}$, and $\pm 12.5\;\mathrm{Nm}\;T_{z}$ with the total accuracy less than ≤1% F.S.

### 5.1. Simulation Study

**Figure 7:**
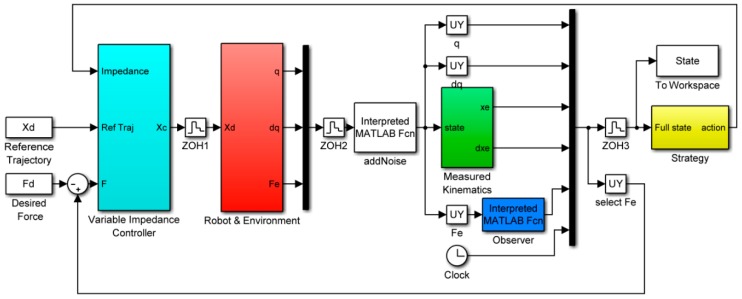
The block diagram of simulation in MATLAB Simulink.

We first evaluated our system through a simulation of force control using MATLAB (R2015b Version 8.6) Simulink. The block diagram of simulation is shown in Figure 7. In the simulation setup, a stiff contact plane is placed under the end-effector of the robot. The stiffness and damping of the plane are set 5000 N/m and 1 Ns/m, respectively. The robot’s base is located at [0, 0, 0] m while the original position of the plane $O_{P}$ is located at [0.2,−0.5,0.15] m. The plane’s length, width, and height are 0.9, 1, and 0.2 m, respectively. The robot should automatically learn to control the contact force to the desired value.

In the simulation, the episode length is set as T=1 s. The control period of the impedance controller is 0.01 s, and the calculation period of the learning algorithm is 0.01 s. The number of total learning iterations, excluding the random initialization, is N=20. The training inputs of the learning algorithm are the position and contact force of the end-effector $x=\left[X,Y,Z,F_{x},F_{y},F_{z}\right]\in {\mathbb{R}}^{6}$. The training targets of the learning algorithm are the desired position and the desired contact force $y=\left[X_{d},Y_{d},\;Z_{d},F_{dx},\;F_{dy},F_{dz}\right]=\left[0.41,\;0,\;0.2265,\;0,\;0,\;15\right]\in {\mathbb{R}}^{6}$. The desired position is the initial position of the end-effector in Cartesian space. The desired contact force in *z*-axis direction is 15 N. If the steady state error of contact force $\left|F_{z}-F_{zd}\right|\leq 1\;N$ and the overshoot is less than 3 N, the task is successful; otherwise, it is failed. The target state in cost function is $x_{target}=\left[0.41,\;0,\;\;0.2265,\;\;0,\;0,\;15\right]$. The strategy outputs are the impedance parameters $u=\left[K_{d}\;B_{d}\right]$. The number of the GP controller is n=10. The ranges of the impedance parameters are set as $K_{d}\in \left[0.1\;25\right]$ and $B_{d}\in \left[50\;1000\right]$. The action penalty coefficient of the cost function is ζ=0.03. The measurement noise of the joint position, joint velocities and the contact force are set as $\delta \sim N\left(0,0.01^{2}\right)$ respectively. In the initial trial, the impedance parameters are initialized to stochastic variables that are subject to $N(u_{0}|0.1u_{max},0.1u_{max})$.

### 5.2. Simulation Results

**Figure 8:**
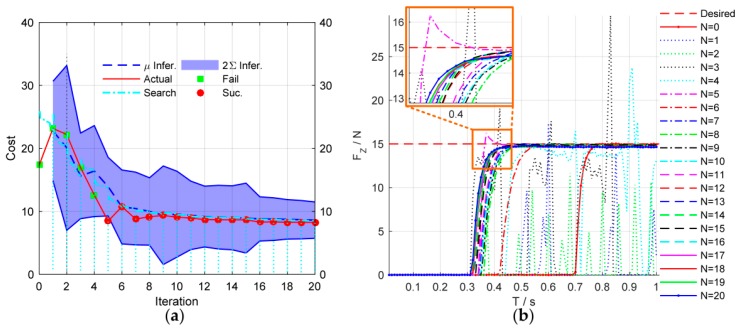
Simulation results of force control learning system. (**a**) The cost curve of learning process; the blue dotted line and the blue shade are the predicted cost mean and the 95% confidence interval. (**b**) The performances of force control during learning process.

**Figure 9:**
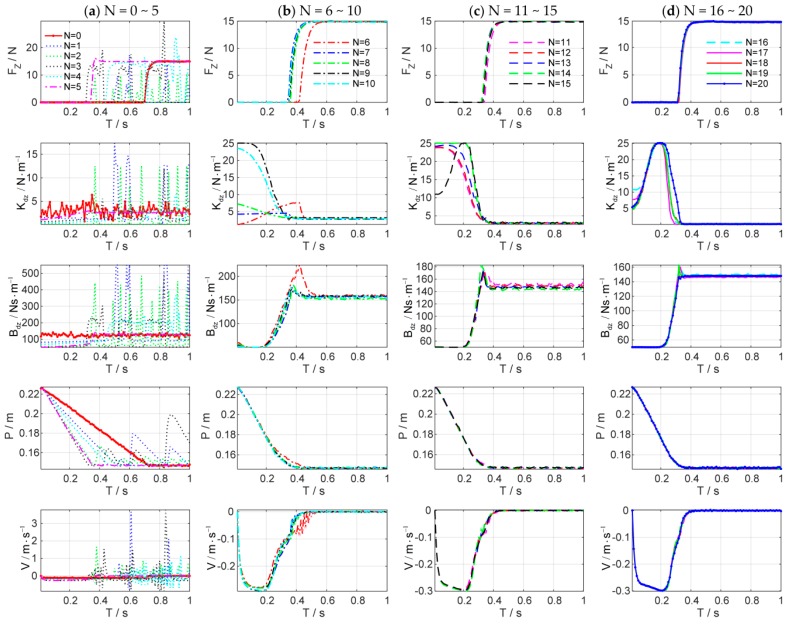
Learning process of the total 20 learning iterations. The first row: contact force in *z*-axis direction; the second row: Cartesian stiffness schedules; the third row: Cartesian damping schedules; the fourth row: position of the end-effector in *z*-axis direction; the fifth row: velocity of the end-effector in *z*-axis direction.

**Figure 10:**
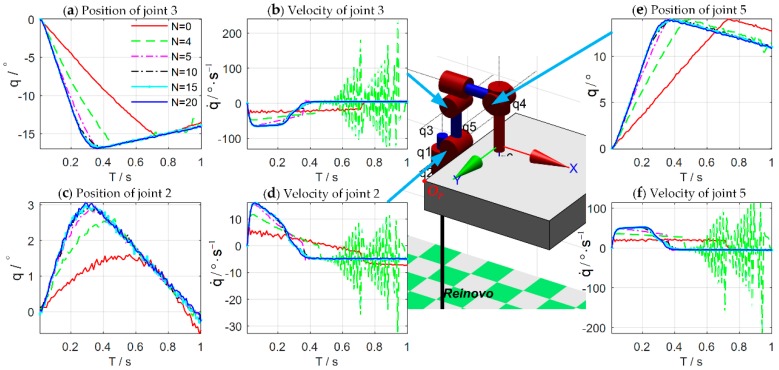
Joint trajectories after 4, 5, 10, 15, and 20 updates for the second, third, and fifth joint of the Reinovo robot.

Figure 8 shows the simulation results of the force control learning system. The block marks in Figure 8a indicate whether the task is successful or not. The light blue dash-dotted line represents the cumulative cost during the explorations. Figure 9 details the learning process of the total 20 learning iterations. Figure 10 shows the joint trajectories after 4, 5, 10, 15, and 20 updates. Note that N=0 denotes the initial trial, and the whole learning process is implemented automatically.

In the initial trial, the robot moves down slowly to search the contact plane and the end-effector begins to contact the plane at T=0.7 s. Due to the large overshoot of the contact force, the task is failed until the fifth learning iteration. After the fifth learning iteration, the end-effector contacts the plane at T=0.34 s, which is faster than the initial trial. The contact force reaches to the desired value rapidly with a little overshoot. The learning algorithm optimizes the control strategy continuously to reduce the cumulative cost. After seven iterations, the strategy’s performance is stable, and the contact force reaches the desired value quickly without overshoot by adjusting the impedance parameters dynamically. The optimal strategy is learned after 20 iterations, and its cumulative cost is the smallest. The end-effector can contact the plane at T=0.3 s.

**Figure 11:**
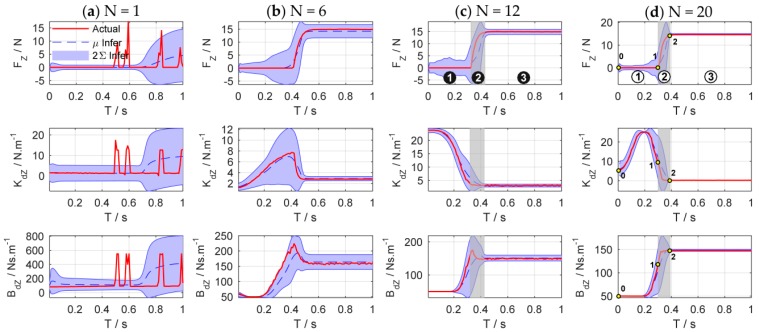
States evolution of force control. Columns (**a**–**d**) are the state evolutions of the 1st, 6th, 12th, and 20th learning iteration, respectively. The top row is the change of contact force $F_{z}$, the second row is the target stiffness $K_{dz}$, and the third row is the target damping $B_{dz}$.

Figure 11 shows the evolutions of force control and impedance profiles. The state evolutions predicted by the GP model can be represented by the blue dotted line and the blue shade, which denote the mean and the 95% confidence interval of the state prediction respectively. The historical sampled data are constantly enriched with the increase of interaction time, and the learned GP model is optimized and stabilized gradually. When a good GP model is learned, it can be used as a faithful proxy of the real system (Figure 11b–d).

As shown in Figure 11d, the process of impedance regulation can be divided into three phases: (1) the phase before contacting the plane; (2) the phase of coming into contact with the plane; and (3) the stable phase of contact with the plane. When the end-effector contact the environment, the manipulator is suddenly converted from free space motion to constrained space motion, and the collision is inevitable. The stiffness of the environment increases suddenly. Consequently, the stiffness of the controller declines rapidly to make the system ‘soft’ to ensure safety. Meanwhile, the damping continues to increase to make the system ‘stiff’ to suppress the impact of environmental disturbance.

Throughout the simulation, only five interactions (5 s of interaction time) are required to learn to complete the force tracking task successfully. Besides, the impedance characteristics of the learned strategy are similar to that of humans for force tracking. The simulation results verify the effectiveness and the efficiency of the proposed system.

### 5.3. Experimental Study

**Figure 12:**
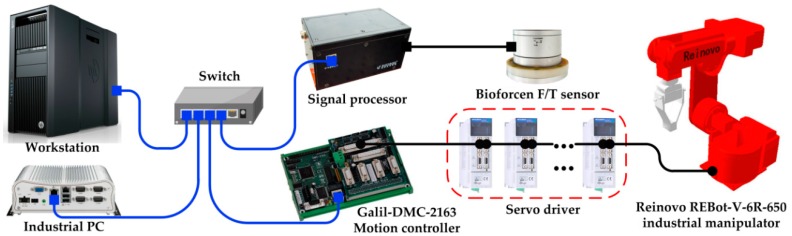
Hardware architecture of the system.

**Figure 13:**
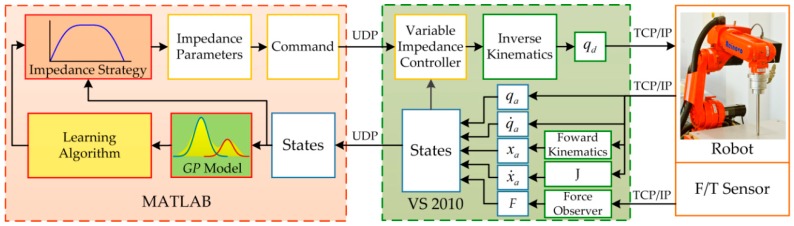
Implementation diagram of the algorithm.

The hardware architecture of the system is shown in Figure 12. The test platform consists of six parts, an industrial manipulator, servo driver, Galil-DMC-2163 motion controller, Bioforcen F/T sensor, an industrial PC, and a workstation. All parts communicate with others through the switch. The motion controller communicates with the industrial PC through TCP/IP protocol running at 100 Hz. The sensor communicates with the industrial PC via Ethernet interface through TCP/IP and samples the data at 1 kHz. The specific implementation diagram of the algorithm is shown in Figure 13. The motion control of the robot is executed using Visual Studio 2010 on the industrial PC. The learning algorithm is implemented by MATLAB on the workstation. The workstation communicates with the industrial PC through UDP protocol.

**Figure 14:**
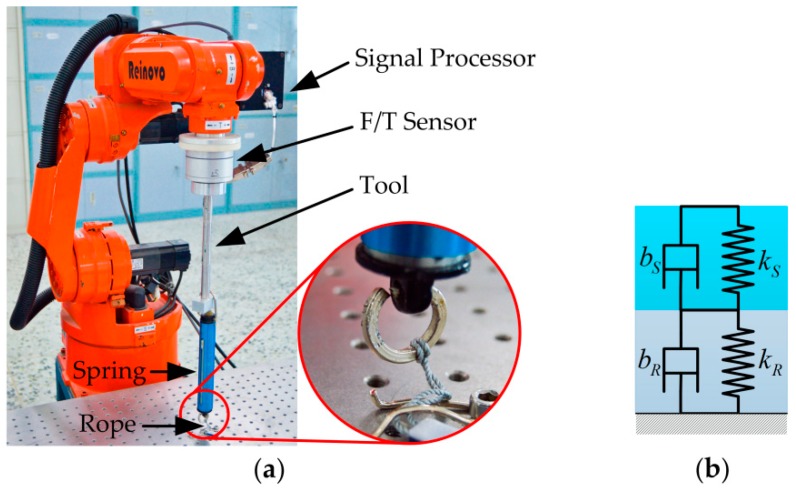
(**a**) Experimental setup; (**b**) simplified model of the contact environment.

To imitate the nonlinear variable characteristics of the circumstance during the force tracking task, a combination of spring and rope is taken as the unstructured contact environment. As shown in Figure 14, the experimental setup mainly consists of a spring dynamometer attached to the tool at the end-effector and a rope of unknown length tied to the spring with the other end fixed on the table. The contact force is controlled by stretching the rope and the spring. Here, the specifications of the rope are unknown, and the rope is in a natural state of relaxation. The measurement range, length, and diameter of the spring dynamometer are 30 Kg, 0.185 m, and 29 mm, respectively. The exact values of the stiffness and damping of the springs are unknown to the system. In the experiments, the episode length (i.e., the prediction horizon) is T=3 s. Other settings are consistent with those of simulations in Section 5.1.

### 5.4. Experimental Results

**Figure 15:**
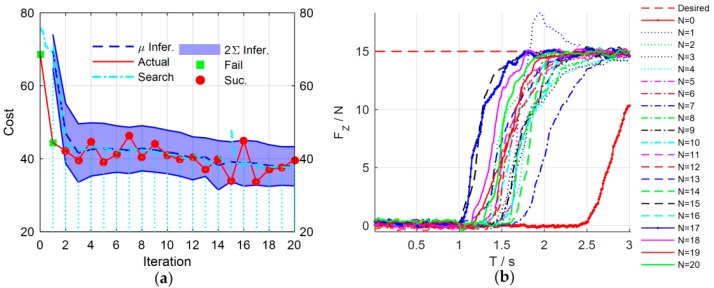
Experimental results of force control learning system. (**a**) The cost curve of learning process. (**b**) The performances of force control during learning process, including a total 20 learning iterations throughout the experiment.

**Figure 16:**
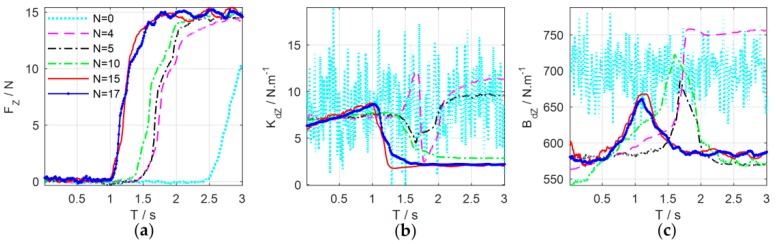
Main iterations of the learning process. (**a**) Contact force; (**b**) Cartesian stiffness schedules; (**c**) Cartesian damping schedules.

The experimental results are shown in Figure 15. Figure 16 details main iterations of the learning process. From the experimental results, we can see that in the initial trial, the manipulator moves slowly and the rope begins to be stretched at T=2.5 s to increase the contact force. The contact force reaches 10.5 N at the end of the test, which implies that the task failed. In the second trial, the rope and the spring can be stretched at T=1.5 s, which is faster than that of the first trial, and the contact force reaches the desired values rapidly, but the task failed because the overshoot is greater than 3 N. Only two learning iterations are needed to complete the task successfully. After 17 iterations, the cumulative cost is the smallest and the force control performance is also the best. The rope can be stretched at T=1 s, and the overshoot is suppressed effectively.

**Figure 17:**
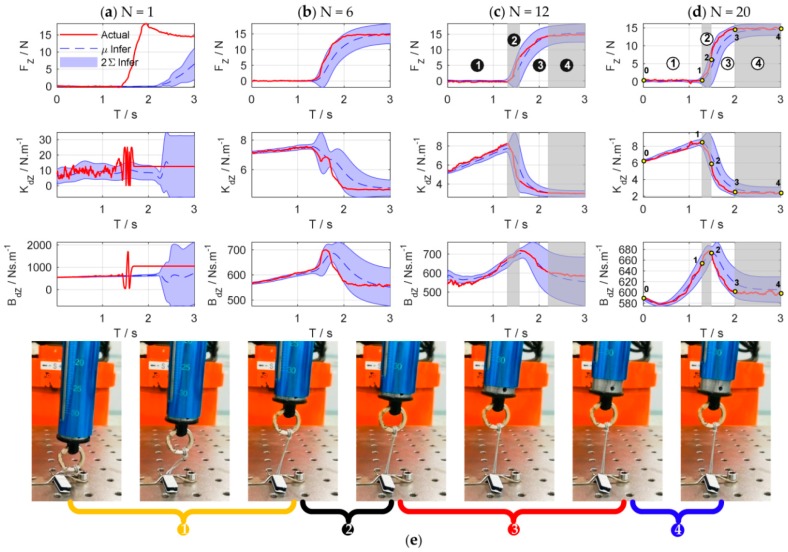
States evolution of force control. Columns (**a**–**d**) are the state evolutions of the 1st, 6th, 12th, and 20th learning iteration, respectively. The top row shows the contact force $F_{z}$, the second row shows the profile of stiffness $K_{dz}$, and the third row shows the profile of damping $B_{dz}$.

**Figure 18:**
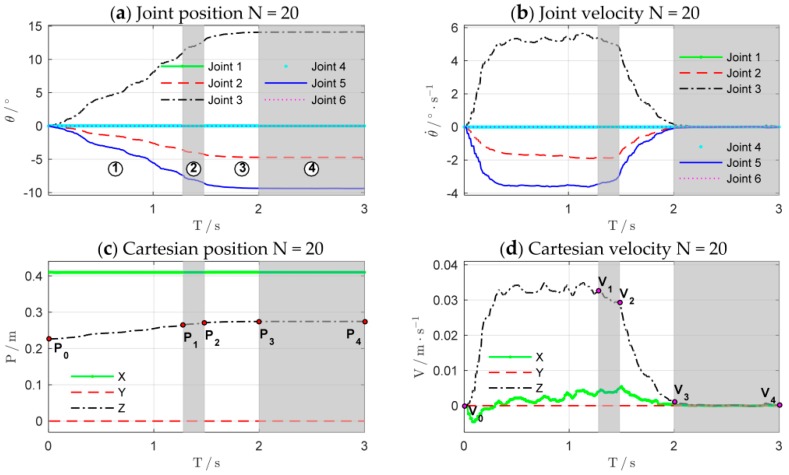
Trajectories during the 20th experiment iteration. (**a**) Joint position; (**b**) Joint velocity; (**c**) Cartesian position of the end-effector; (**d**) Cartesian velocity of the end-effector.

Figure 17 shows the evolutions of force control and impedance profiles. The bottom row shows the stretching process of the combination of the rope and the spring. The joint trajectories and Cartesian trajectories during the 20th experiment iteration are shown in Figure 18. The trajectories of other iterations are similar to those of the 20th iteration. The stretching process of the combination can be divided into four phases, just as the shaded areas shown. Table 1 summarizes the key states of the four phases. The corresponding subscripts of $F_{z}$, $K_{dz}$, and $B_{dz}$ are shown in Figure 17d while the subscripts of position (P) and velocity (V) are shown in Figure 18c,d.

The four phases of impedance regulation are corresponded to the force control process mentioned above:

1. $T_{0}-T_{1}$: Phase before stretching the rope. The manipulator moves freely in the free space. To tighten the rope quickly, the movement of the end-effector increases as the impedance increase. The contact force is zero in this phase.
2. $T_{1}-T_{2}$: Phase of stretching the rope. When the rope is stretched, the manipulator is suddenly converted from free space motion to constrained space motion. The stiffness of the environment increases suddenly, and this can be seen as a disturbance of environment. Consequently, the stiffness of the controller declines rapidly to make the system ‘soft’ to ensure safety. Meanwhile, the damping continues to increase to make the system ‘stiff’ to suppress the impact of environmental disturbance and avoid oscillation. On the whole, the system achieves an appropriate strategy by weighting ‘soft’ and ‘stiff’. In this phase, the contact force increases rapidly until the rope is tightened.
3. $T_{2}-T_{3}$: Phase of stretching the spring. The spring begins to be stretched after the rope is tightened. Although the environment changes suddenly, the controller does not select the strategy as Phase 2; it makes the system ‘soft’ by gradually reducing the stiffness and damping to suppress the disturbances. In this way, the contact force increases slowly to avoid overshoot when approaching the desired value.
4. $T_{3}-T_{4}$: Stable phase of stretching the spring. The manipulator contacts with the environment continuously and the contact force is stabilized to the desired value. In this phase, the stiffness and damping of the controller are kept at minimum so that the system maintains the ability of compliance.

There are total 20 learning iterations throughout the experiment. In the early stage of learning, the uncertainties of the GP model are large due to the lack of collected data. With the increase of interaction time, the learned GP model can be improved gradually. After two learning iterations, which means that only 6 s of interaction time is required, a sufficient dynamic model and strategy can be learned to complete the force tracking task successfully. The experimental results above verify that the proposed force control learning system is data-efficient. It is mainly because the system explicitly establishes the transition dynamics that are used for internal virtual simulations and predictions, and the optimal strategy is improved by evaluations. In this way, more efficient information could be extracted from the sampled data.

### 5.5. Comparative Experiments

#### 5.5.1. Environmental Adaptability

**Figure 19:**
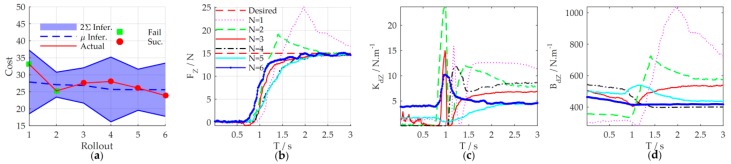
Experimental results of environmental adaptability. (**a**) Cost curve; (**b**) contact force; (**c**) Cartesian stiffness schedules; (**d**) Cartesian damping schedules.

The results above have verified that the learned strategy could adjust the impedance profiles to adapt to the environmental change in the episodic case. However, what happens if the whole contact environment changes? In order to verify the environmental adaptability of the proposed learning variable impedance control method, we use another different spring dynamometer to build the unstructured contact environment. The measurement range, length, and diameter of the second spring dynamometer are 15 Kg, 0.155 m, and 20 mm, respectively. It implies that the stiffness and location of the environment are all changed. Other experimental setups are consistent with the Section 5.3. The initial strategy for the second environment is the learned (sub)optimal strategy for the first spring (*N* = 17 in Section 5.4). The results are illustrated in Figure 19.

From the experimental results, we can see that in the initial application of the control strategy, even though the impedance profile is regulated online according to the learned strategy, the task is failed. This is mainly because the whole environment is changed a lot, the strategy learned for the previous environment is not suitable for current environment. However, as the learning process continues to optimize, the learned (sub)optimal strategy could adapt to the new environment and successfully complete the task after two learning iterations. Therefore, the proposed method could adapt to new environments, taking advantage of its learning ability.

#### 5.5.2. Comparison of Force Control Performance

**Figure 20:**
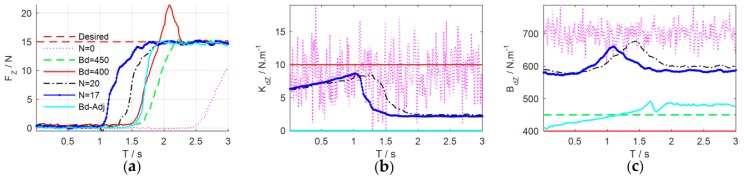
Experimental comparison results. (**a**) Force control performance; (**b**) target stiffness; (**c**) target damping.

Variable impedance control can regulate the task-specific impedance parameters at different phases to complete the task more effectively, which is the characteristic that the constant impedance control is not equipped. Next, the proposed learning variable impedance control is compared with the constant impedance control and the adaptive impedance control [13] to verify the effectiveness of force control. The stiffness of the constant impedance controller is set as $K_{d}=10$ while the stiffness of the adaptive impedance controller is set as $K_{d}=0$ [13]. The damping of the controller could be adjusted manually or automatically. Experimental comparison results are illustrated in Figure 20. 

In order to quantitatively compare the performances, we use four indicators to quantify the performances. The first indictor is the time $T_{free}$ when the force begins to increase, i.e., the movement time of the robot in free space. The second indicator is the accumulated cost $J^{\pi }\left(\theta \right)$ which is defined in Equation (33). Note that the accumulated cost includes two parts: the cost caused by the error to the target state (Equation (36)) and the cost caused by the control gains (Equation (37)). The third one is the root-mean-square error (*RMSE*) of the contact force

(44) $$RMSE=\sqrt{\frac{\sum_{t=1}^{H}{\left(F_{z}(t)-F_{zd}\right)}^{2}}{H}},$$

where $F_{z}\left(t\right)$ is the actual contact force in *Z*-axis direction, and $F_{zd}$ is the desired contact force. H is the total number of the samples during the episode.

An energy indicator is defined to indicate the accumulated consumed energy during the control process

(45) $$Ey=\sum_{t=1}^{H}\sum_{i=1}^{6}\frac{1}{2}m_{i}{\dot{\theta }}_{i}^{2}(t),$$

where $m_{i}$ is the mass of the $i^{th}$ joint and ${\dot{\theta }}_{i}$ is the angular velocity of the $i^{th}$ joint. Actually, the masses of the joints are unknown accurately. Without loss of generality, the masses can be approximately set as $\left[m_{1},\;m_{2},\;m_{3},\;m_{4},\;m_{5},\;m_{6}\right]=\left[1,\;1,\;1,\;0.5,\;0.3,\;0.1\right]$.

Table 2 reveals the performance indicators of the three impedance controllers. Compared with the constant/adaptive impedance control, the learning variable impedance control has the minimum indicator values, which indicates that the proposed system is effective. Obviously, the performance of the adaptive impedance control is better than the constant one, but still worse than the optimal impedance strategy learned by the proposed learning variable impedance control.

#### 5.5.3. Learning Speed

**Figure 21:**
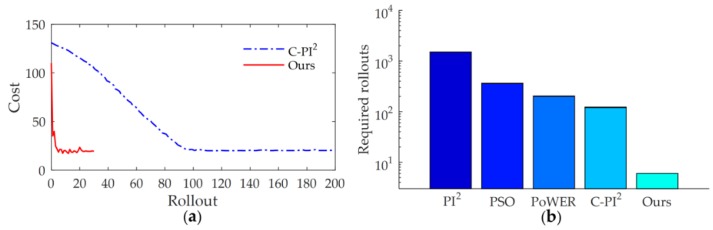
(**a**) The cost curve of the learning process. (**b**) Comparison of learning speed with other learning variable impedance control methods.

In order to further illustrate the efficiency of the proposed method, we compared the learning speed with state-of-the-art learning variable impedance control method, C-PI^2^, through the via-gain task [37]. The cost function of C-PI^2^ is chosen as $r_{t}=w_{1}\delta \left(t-0.4\right)\|K_{1}-K_{t}^{P}\|$ with $w_{1}=1\times 10^{8}$ $K_{1}=15$. The cost function of our method is chosen as the Equation (35) with $x_{target}=15$, ζ=0.03. The cost curve of learning process is shown in Figure 21a. Similar to other studies [37], to indicate the data-efficiency of the method, we take the required rollouts to get the satisfactory strategy as the indicator of the learning speed. The results show that C-PI^2^ converges after about 92 rollouts, whereas our method needs only 4 rollouts.

Current learning variable impedance control methods usually require hundreds or thousands of rollouts to get a stable strategy. For tasks that are sensitive to the contact force, too many physical interactions with the environment during the learning process are often infeasible. Improving the efficiency of learning method is critical. Figure 21b shows the comparison of learning speed with other learning variable impedance control methods. From the results of [4,37,50], we can see that, to get a stable strategy, PI^2^ needs more than 1000 rollouts, whereas PSO requires 360 rollouts. The efficiency of PoWER is almost the same as that of C-PI^2^, which requires 200 and 120 rollouts, respectively. The learning speed of C-PI^2^ is much higher than that of previous methods but is still slower than our method. Our method outperforms other learning variable impedance control methods by at least one order of magnitude.

## 6. Discussion

According to the definition of the cost function (35)–(37), we can learn that decreasing the distance between $x_{t}$ and $x_{target}$ and keeping the impedance parameters $u_{t}$ at a low level will be beneficial for minimizing the accumulated cost. Consequently, small damping parameters will make the robot move quickly to contact with the environment to reduce the distance between $x_{t}$ and $x_{target}$. Unfortunately, small damping could reduce the positioning accuracy of the robot and thus make the system with poor ability to suppress disturbances. On the contrary, large damping could improve the system’s ability of suppressing disturbances and reduce the speed of motion. It will lead to task failure if the impedance parameters cannot be regulated to suppress the overshoot. Hence, the learning algorithm must make a tradeoff between rapidity and stability to achieve the proper control strategy. By regulating the target stiffness and damping independently at different phases, the robot achieves rapid contact with the environment while the overshoot is effectively suppressed.

The impedance characteristics of the learned strategy are similar to the strategy that employed by humans for force tracking. Reduce the impedance by muscle relaxation to make the system ‘soft’ when it needs to guarantee safety, while increase the impedance by muscle contraction to makes the system ‘stiff’ when it needs to guarantee fast-tracking or to suppress disturbances. When the contact environment is stable, the arm is kept in a compliant state by muscle relaxation to minimize the energy consumption. Our system learns the optimal impedance strategy automatically through continuous explorations. This is different from the methods of imitating human impedance behavior, such as [18] and [24], which usually need additional device—e.g., EMG electrodes—to transfer human skills to the robots by demonstration.

**Figure 22:**
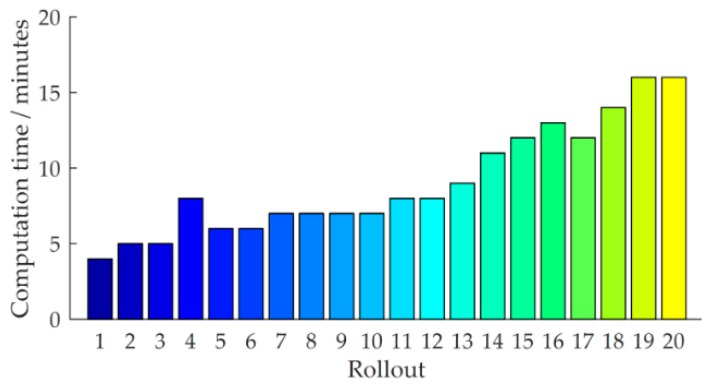
Computational time for each rollout.

The proposed learning variable impedance control method emphasizes the data-efficiency, i.e., sample efficiency, by learning a GP model for the system. This is critical for learning to perform force-sensitive tasks. Note that only the application of the strategy requires physical interacting with the robot; internal simulations and strategy learning only use the learned GP model. Although a fast converging controller is found, the proposed method still has the computation intensive limitation. The computational time for each rollout on a workstation computer with an Intel(R) Core(TM) i7-6700 K CPU@4.00 GHz is detailed in Figure 22.

Obviously, between the trials the method requires approximately 9 min to find the (sub)optimal strategy. With the increase of sample data set, the computational time is increasing gradually, because the kernel matrices need to be stored and inverted repeatedly. The most demanding computations are the predictive distribution and the derivatives for prediction using the GP model.

## 7. Conclusions and Future Works

In this paper, we presented a data-efficient learning variable impedance control method that enables the industrial robots automatically learn to control the contact force in the unstructured environment. The goal was to improve the sampling efficiency and reduce the required physical interactions during learning process. To do so, a GP model was learned as the faithful dynamics of the system, which is then used for internal simulations to improve the data-efficiency by predicting the long-term state evolution. This method learned an impedance regulation strategy, based on which the impedance profiles were regulated online to track the desired contact force. In this way, the flexibility and adaptivity of the system were enhanced. It is worth noting that the optimal impedance control strategy, which is equipped with the similar impedance characteristics of humans, is automatically learned through several iterations. There is no need to transfer human skills to the robot with additional sampling devices. The effectiveness and data-efficiency of the system were verified through simulations and experiments on the six-DoF Reinovo industrial robot. The learning speed of this system outperforms other learning variable impedance control methods by at least one order of magnitude.

Currently, the described work only focuses on the efficient learning of force control. In the future work, we will extend this system to learn to complete more complex tasks that are sensitive to contact force, such as assembly tasks of fragile components. Furthermore, parallel and online implementation of this method to improve computational efficiency would be a meaningful and interesting research direction.

## Figures and Tables

**Table 1:** Key states of the four phases during the 20th iteration.

Subscript	T/s	P/m	$V/m\cdot s^{-1}$	$F_{z}/N$	$K_{dz}/N\cdot m^{-1}$	$B_{dz}/Ns\cdot m^{-1}$
0	0.00	0.2265	0.0000	0.393	6.246	588.97
1	1.28	0.2642	0.0327	0.326	8.468	653.99
2	1.48	0.2701	0.0292	6.095	5.888	673.83
3	2.00	0.2744	0.0010	14.625	2.469	601.93
4	3.00	0.2745	0.0000	14.874	2.379	598.53

**Table 2:** Performance indicators.

Mode	Name	$T_{free}$/s	Cost	RMSE	$Ey\;\left(\times 10^{3}\right)$	Overshoot
Constant Impedance control	Bd = 450	1.70	43.29	11.29	5.51	No
	Bd = 400	1.50	41.09	10.83	5.85	Yes
Adaptive Impedance control	Bd-Adj	1.50	37.23	11.09	5.38	No
Learning variable Impedance control	N = 20	1.25	39.71	10.19	2.33	No
	N = 17	1.00	33.64	9.23	2.08	No
